# Synergistic interactions in multispecies biofilm combinations of bacterial isolates recovered from diverse food processing industries

**DOI:** 10.3389/fmicb.2023.1159434

**Published:** 2023-04-13

**Authors:** Faizan Ahmed Sadiq, Koen De Reu, Mette Burmølle, Sharon Maes, Marc Heyndrickx

**Affiliations:** ^1^Technology and Food Science Unit, Flanders Research Institute for Agriculture, Fisheries and Food (ILVO), Merelbeke, Belgium; ^2^Section of Microbiology, Department of Biology, University of Copenhagen, Copenhagen, Denmark; ^3^The Department of Ecotechnology and Sustainable Building Engineering, Mid Sweden University, Östersund, Sweden; ^4^Department of Pathobiology, Pharmacology and Zoological Medicine, Ghent University, Merelbeke, Belgium

**Keywords:** bacterial interactions, synergy, dairy industry, biofilms, disinfection

## Abstract

Most biofilms within the food industry are formed by multiple bacterial species which co-exist on surfaces as a result of interspecies interactions. These ecological interactions often make these communities tolerant against antimicrobials. Our previous work led to the identification of a large number (327) of highly diverse bacterial species on food contact surfaces of the dairy, meat, and egg industries after routine cleaning and disinfection (C&D) regimes. In the current study, biofilm-forming ability of 92 bacterial strains belonging to 26 genera and 42 species was assessed and synergistic interactions in biofilm formation were investigated by coculturing species in all possible four-species combinations. Out of the total 455 four-species biofilm combinations, greater biofilm mass production, compared to the sum of biofilm masses of individual species in monoculture, was observed in 34 combinations. Around half of the combinations showed synergy in biofilm mass > 1.5-fold and most of the combinations belonged to dairy strains. The highest synergy (3.13-fold) was shown by a combination of dairy strains comprising *Stenotrophomonas rhizophila*, *Bacillus licheniformis*, *Microbacterium lacticum*, and *Calidifontibacter indicus*. The observed synergy in mixed biofilms turned out to be strain-specific rather than species-dependent. All biofilm combinations showing remarkable synergy appeared to have certain common species in all combinations which shows there are keystone industry-specific bacterial species which stimulate synergy or antagonism and this may have implication for biofilm control in the concerned food industries.

## Introduction

Biofilms are multicellular surface-associated microbial communities embedded in a self-produced or shared matrix containing a wide array of extracellular polymeric substances (EPS) (Karygianni et al., 2020). Microbial biofilms are abundantly present as the prevalent form of microbial life in almost all industrial, clinical, and natural systems with substantial impact on human and animal health, agriculture, food processing, water security, marine sector, and many other industrial processes.

Biofilms on food and non-food contact surfaces pose food safety and quality challenges in food industries and the control of microbial biofilms in the food industry from farm-to-fork costs 324 billion USD globally (Highmore et al., 2022). Microorganisms comprising biofilms in the food production facility include both spoilage and pathogenic bacteria (Nan et al., 2022) which often co-exist as a result of intricate interactions, mainly due to metabolic dependencies (i.e., auxotrophies) and community-level benefits, including protection from antimicrobials (Sadiq et al., 2021a; Wit et al., 2022) and predator evasion (Goh et al., 2023). Surfaces in the food industry are routinely subjected to cleaning and disinfection (C&D) processes since the presence of biofilms is constantly detected on surfaces. Bacteria in mixed biofilm communites can survive C&D treatments on food contact surfaces either due to protective effects of the biofilm matrix (self-produced or shared) or due to inter-species interactions among the community members in which resistant strains often provide high levels of antibiotic protection to otherwise sensitive strains by inducing genetic or environmental changes in their favor (Parijs and Steenackers, 2018; Bottery et al., 2022). Biofilms are also an important concern for the food industry because the microenvironments created within the biofilm structure may be conducive to higher production of spoilage enzymes compared to the planktonic state of bacteria (Teh et al., 2014).

Different species co-existing in a biofilm microenvironment influence the growth, abundance, and physiological characteristics of other species through several interactions including positive (mutualism, cooperation, syntrophy, synergism, and altruism) as well as exploitative and antagonistic interactions (Nadell et al., 2016). Microbial species secrete numerous enzymes, signaling and scavenging molecules that can affect the growth and survival of other microbial cells in their vicinity (Liu et al., 2017). Sometimes there are keystone species in a biofilm community which – regardless of their proportion – are often involved in promoting biofilm formation in other species as well as protecting other community members from external stressors (Parijs and Steenackers, 2018; Karki et al., 2021).

Despite the development of a large number of chemical, physical, and biological approaches to control biofilms in the food industry (Coughlan et al., 2016; Mevo et al., 2021), still in several cases no clear solution seems to exist to deal with the risk biofilms pose. An important aspect that is yet less explored in biofilms in the food industry is interspecies interactions. There is convincing evidence that interspecies social behavior of bacteria, especially metabolic cross-feeding, influence resistance development against antimicrobials (Adamowicz et al., 2018). Thus, cataloging community composition on food contact surfaces and exploring overall interactions between community members are vital to further develop biofilm monitoring and controlling strategies. Many studies on microbiota of food contact surfaces have only focused on microbial ecology of surfaces with less focus on underlying biological interactions (Bridier et al., 2019; Wagner et al., 2020; Fagerlund et al., 2021). Existing studies on bacterial interactions in mixed-species biofilms are mainly on bacterial species originated from oral biofilms (Kommerein et al., 2018; Wang et al., 2020), soil (Ren et al., 2015) or the human gut (Sadiq et al., 2021b). There is some evidence that interactions among the co-localized bacterial species on food contact surfaces in mixed biofilms influence biofilm production which has high relevance for food production facilities (Røder et al., 2015; Langsrud et al., 2016; Wang et al., 2021; Yang et al., 2023).

Our group reported a large diversity of bacteria including pathogens and spoilage organisms on food contact surfaces in the dairy, egg, and meat processing facilities after routine C&D (Maes et al., 2019). *Microbacterium* and *Stenotrophomonas* species were identified as the prevalent contaminants in all industries. The aim of this work was to characterize the biofilm-forming potential of the isolates that survive C&D and to find out bacterial species that likely co-exist and persist on food contact surfaces after C&D as a result of synergistic interactions in mixed-species biofilms.

## Materials and methods

### Bacterial strains used in this study

**Figure 1:**
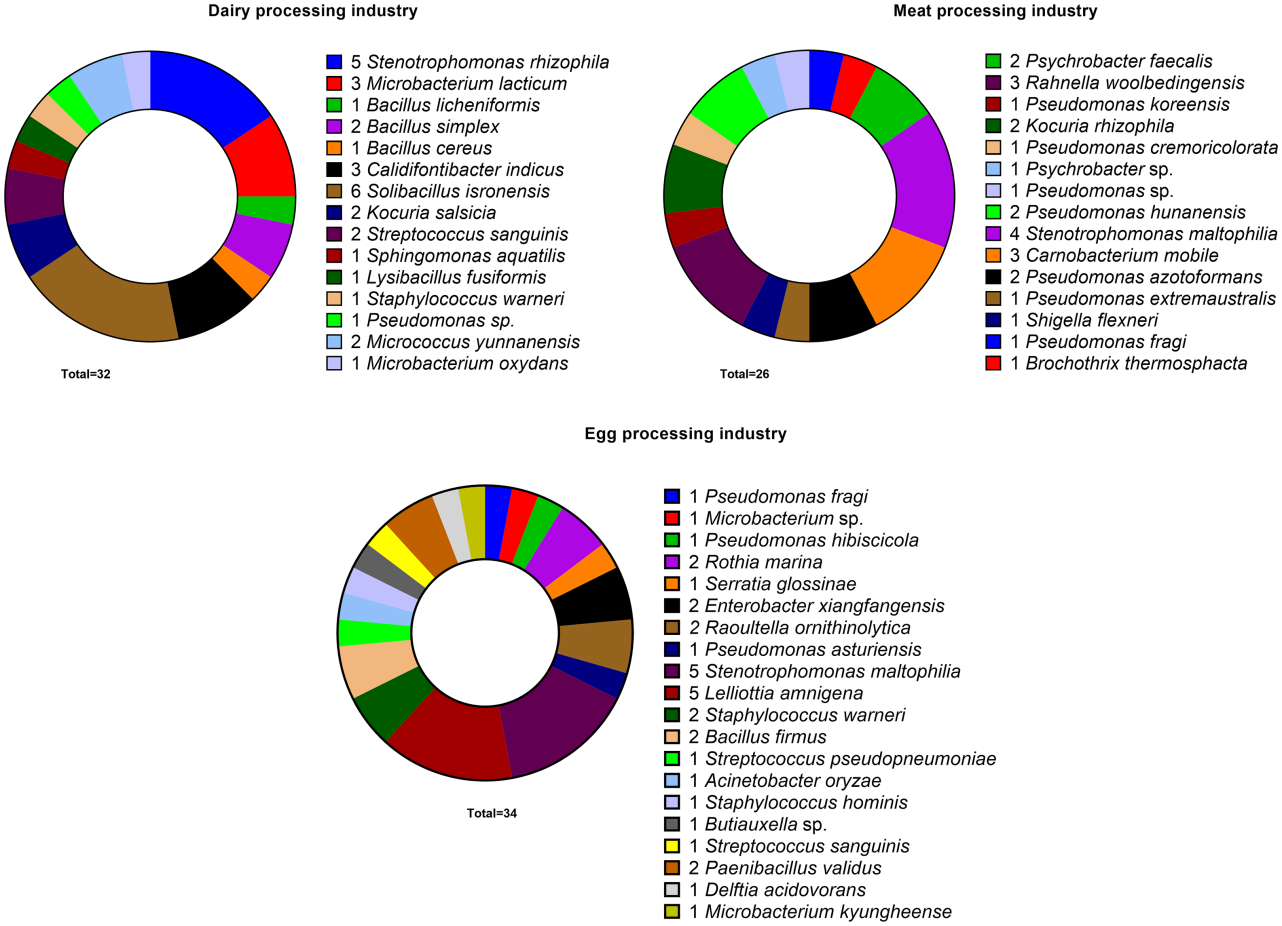
Details concerning number, identification and origin of 92 bacterial strains that were studied for their biofilm forming ability and synergistic interactions in multispecies biofilms. The strains were originated from three food processing industries: dairy, meat, and egg processing industries after routine cleaning and disinfection regimes.

A total of 92 bacterial strains were used in the study which were a part of the previously isolated bacterial strains from food contact surfaces of three food processing industries (dairy, meat, and egg) after routine C&D (Maes et al., 2019). The strains were highly diverse and belonged to 26 genera and 42 different species. Figure 1 shows further details related to the number and identification of these strains with their origin. All strains were grown in a general-purpose medium (Brain-Heart-Infusion, BHI: Merck, Darmstadt, Germany) at 30 or 37°C, depending on the strain and stored at –70°C until this trial. Only two temperatures (30°C and 37°C) were used to determine bacterial optimum growth temperatures. Supplementary Table S1 in Supplementary Material File 1 (sheet 17) contains details of growth temperatures for each bacterial species that were used in biofilm combinations.

### Biofilm quantification using crystal violet staining

Biofilm formation was assayed and quantified as previously described by Ren et al., 2015 using 96-well microtiter plates (Coster 3,596, Corning Inc., Corning, NY, United States). Bacterial strains were incubated overnight for 16–18 h in BHI at their optimum growth temperature (30 or 37°C) followed by appropriate dilution in BHI to the OD_595_ value of 0.05 for all strains. A total of 160 μL was used as the inoculum volume for monospecies biofilms whereas 40 μL for each species was pooled together for four-species biofilm combinations. After 24-h incubation at 30°C or 37°C without shaking, biofilm formation was quantified by measuring the absorbance at 595 nm (Abs_595_) crystal violet (CV) straining. Selection of the temperature of incubation was based on the optimum growth temperature of each species based on preliminary trials of bacterial growth at different temperatures. Strains were classified as non-biofilm formers, weak biofilm formers, moderate biofilm formers and strong biofilm formers based on their OD values. The cutoff OD (ODc) was defined as three SDs above the mean OD of the negative control (Abs_595_: 0.17). Biofilm forming potential was determined as follows: OD ≤ ODc = non-biofilm former, ODc < OD ≤ 2 × ODc = weak biofilm former, 2 × ODc < OD ≤ 4 × ODc = moderate biofilm former and 4 × ODc < OD = strong biofilm former. In the best biofilm combination, some species were also replaced with their other strains to see if the observed synergy is strain-dependent or species-specific. The strains were already well-characterized using pulsed-field gel electrophoresis.

### Grouping of bacterial strains

The 92 strains were divided into 13 different groups with each containing seven different species and all strains in one group were permutated in all possible four-species combinations (35 combinations in each group, 455 in total) to determine synergy in multispecies biofilms. The details concerning species in each group are given in Supplementary File 1 (sheet 4 to 16).

Most of the species in each group were those that were initially recovered from the same food contact surface after C&D (Maes et al., 2019). In addition, species which are known to be prevalent in food industries and are either pathogenic or spoilage in nature were mandatorily included so that their interactions with other species could be identified. For the assessment of synergy, average absorbance of the four-species biofilm (Abs_595_ FS) combination with the sum of absorbances of individual species (Abs_595_ SIS): Abs_595_ FS > Abs_595_ SIS = synergy.

### Scanning electron microscopy

One four-species biofilm combination that showed the highest synergy was developed on stainless steel (SS) coupons (AISI 304 grade: 30 ×15 mm dimension) using 6-well microtiter plates (Coster 3,516, Corning Inc., Corning, NY, USA) by placing the coupons horizontally in each well containing 5 ml BHI followed by incubation at 24 h at the required temperature. All single strains as a part of the combination were also allowed to form biofilm on SS coupon. The coupons were subjected to scanning electron microscopy (SEM) to confirm the presence of all species in the respective combination and to observe any apparent changes in the biofilm morphology. SEM was used to observe biofilms developed on SS coupons for only one combination that showed the highest synergy in a four-species biofilm. Briefly, the coupons were first rinsed with sterile double distilled water and then double fixed with 2.5% glutaraldehyde (Sigma-Aldrich, Saint Louis, Missouri, United States) in 0.1 M sodium cacodylate (Sigma-Aldrich, Saint Louis, Missouri, United States) (pH 7.4) for more than 8 h. The coupons were then post fixed with 1% osmium tetroxide (Sigma-Aldrich, Saint Louis, Missouri, United States) in 0.1 M cacodylate for 1–2 h followed by 3 consecutive washings (5 min each) with 0.1 M cacodylate. Dehydration was performed in graded alcohol solution (30–100% v/v solutions). Finally, the SS coupons were dehydrated with liquid CO_2_ in a Hitachi Model HCP-2 critical point dryer. Hitachi Model E-1010 ion sputter was used to coat the dehydrated samples with gold–palladium for 4–5 min and biofilms were observed in Zeiss Crossbeam 540 FIB-SEM.

### Statistical analysis

Each experiment (mono-species as well as four-species biofilm-forming trials) was repeated three times on different occasions with three replicates. The optical density per strain or four-species combination was measured from 4 wells of each of the three microtiter plates in each trial. Statistical calculations were based on Duncan’s *post hoc* analysis to determine significant differences that were performed using SPSS (SPSS Statistics 23.0) and GraphPad Prism 9. Values of *p* ≤ 0.05 were considered statistically significant.

## Results

### Commonly reported species in three food industries after C&D

Members of some bacterial genera were common among survivors of C&D in all three industries, for instance, bacterial strains belonging to the genera *Bacillus*, *Pseudomonas*, and *Stenotrophomonas* were reported in all industries (Figure 1). *Stenotrophomonas* turned out to be the most prevalent genus among all isolates as 13% of all strains belonged to either *S. rhizophila* or *Stenotrophomonas maltophilia*. *Pseudomonas* was the second most prevalent genus representing 10 strains belonging to 8 different species. Bacterial genera that were commonly present on food contact surfaces of 2/3 industries included *Kocuria*, *Microbacterium*, *Staphylococcus,* and *Solibacillus* in dairy and meat processing, and *Staphylococcus* and *Streptococcus* in dairy and egg processing (Figure 1). Strains collected from the egg processing industries represented more unique species that were not present in other two industries, for example, *Buttiauxella* sp., *Lelliottia amnigena*, *Serratia glossinae*, *Paenibacillus validus*, and *Raoultella ornithinolytica*.

### Biofilm forming ability of the strains and their prevalence

**Figure 2:**
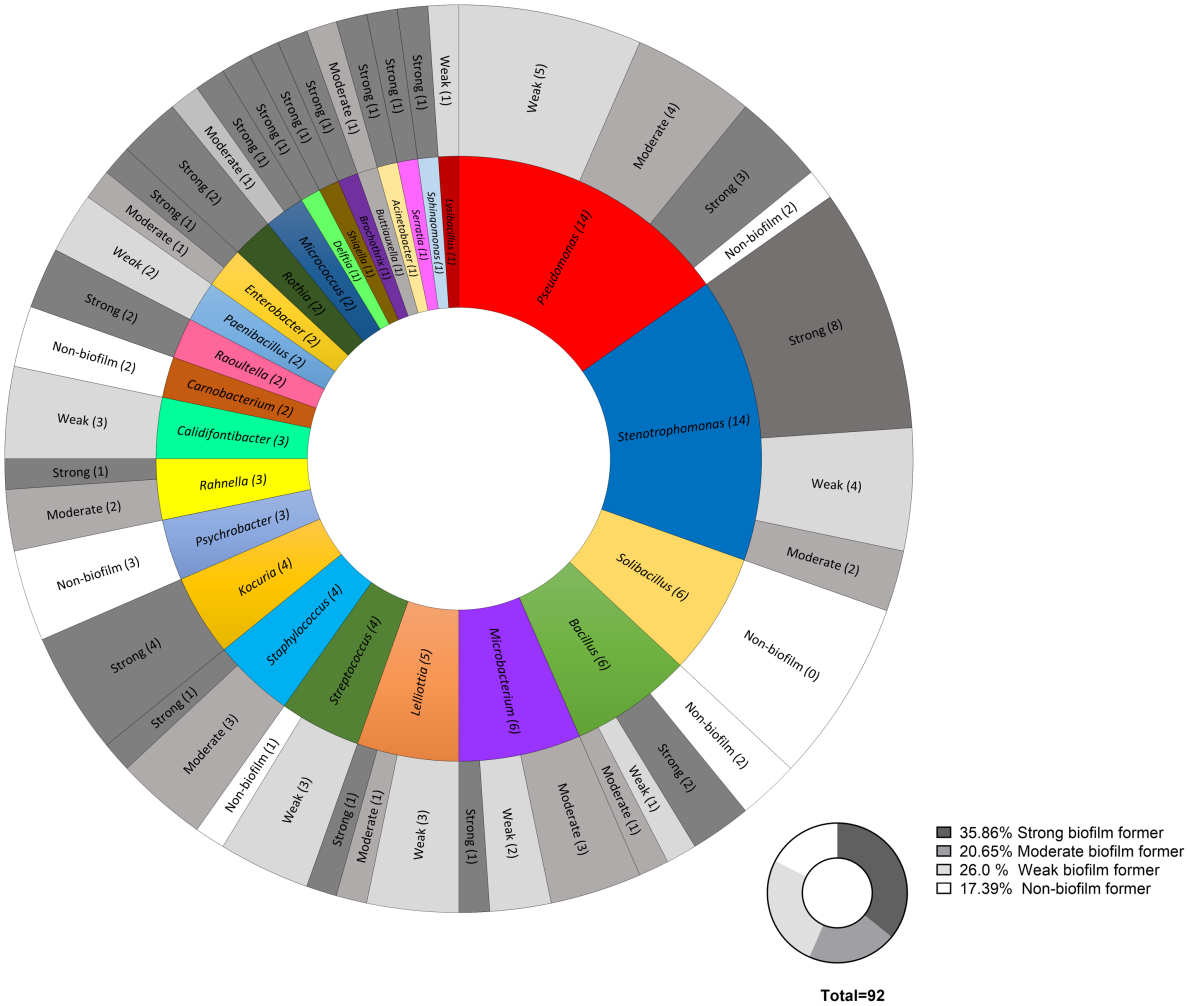
Classification of the biofilm-forming ability of the total strains at the genus level from food contact surfaces of three food industries (dairy, meat, and egg processing) after cleaning and disinfection into non-biofilm formers, weak biofilm formers, moderate biofilm formers, and strong biofilm formers based on their OD values. The cutoff OD (ODc) was defined as three SDs above the mean OD of the negative control (Abs_590_: 0.17). Biofilm forming potential was determined as follows: OD ≤ ODc = non-biofilm former, ODc < OD ≤ (2 × ODc) = weak biofilm former, 2 × ODc < OD ≤ 4 × ODc = moderate biofilm former and 4 × ODc < OD = strong biofilm former.

**Figure 3:**
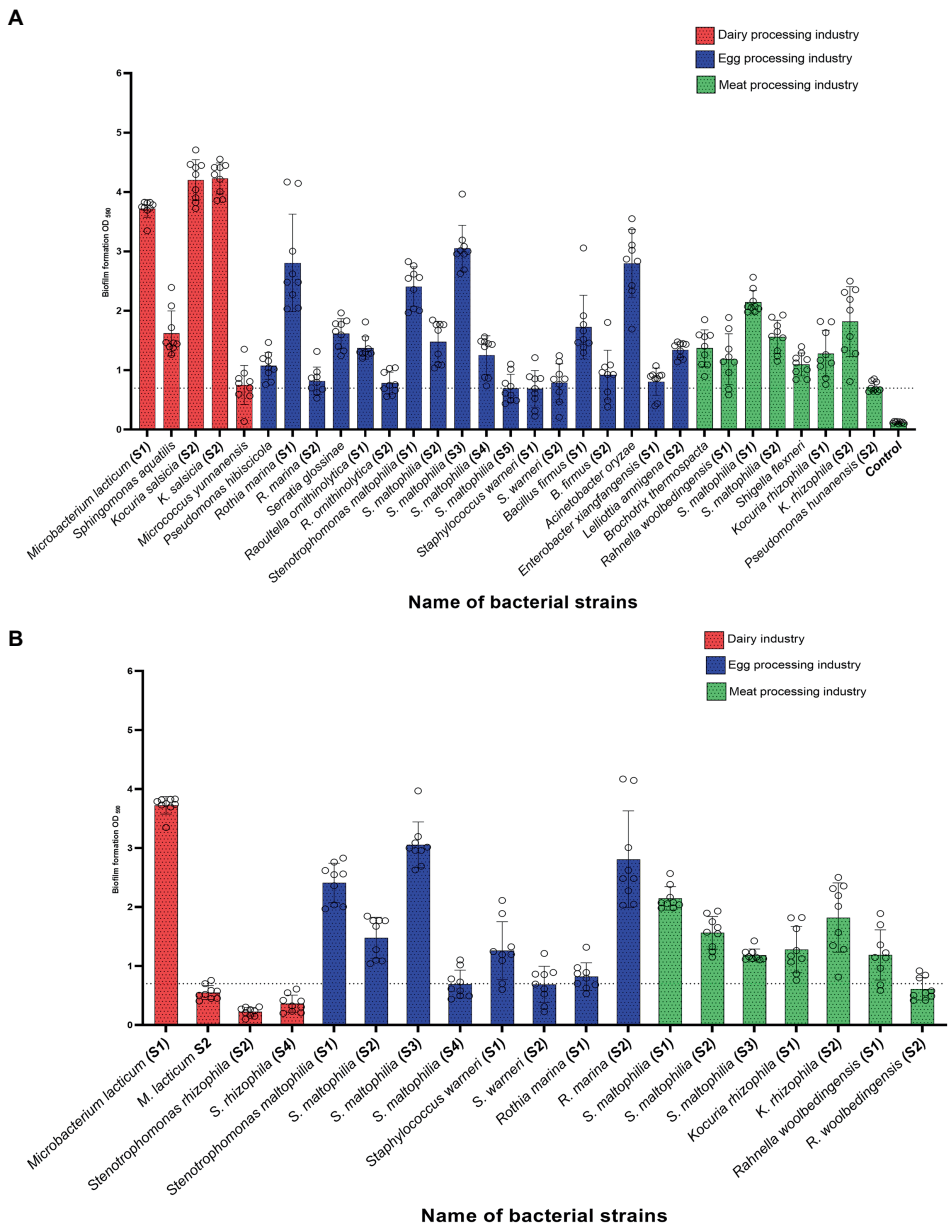
**(A)** High biofilm-forming bacterial strains among 92 isolates collected from food contact surfaces of three industries: dairy, meat, and egg processing industries after routine cleaning and disinfection. **(B)** shows strain-level variation in the biofilm-forming ability of the strains from each industry. The horizontal dotted line in both parts of the figure indicates the OD cut-off value for strong biofilm formation.

A total of 92 bacterial strains, previously recovered from food contact surfaces of three food processing industries after routine C&D, were tested for their ability to form biofilms in single culture on polystyrene surfaces. The strains were classified into four groups: non-biofilm formers, weak biofilm-formers, moderate and strong biofilm formers (Figure 2). Most of the isolates (~ 65%) proved to be either non-biofilm formers, weak or moderate biofilm formers. A total of 33 strains (~ 35%) from all three food industries turned out to be high biofilm formers, and out of which 5 belonged to dairy strains, 8 belonged to the strains collected from the meat processing industry, and 18 strain had originated from the egg processing facility (Figure 3A). The approximate percentage of strong biofilm-formers among the isolates of dairy, meat, and egg processing industries was as follows: 16, 31, and 53%, respectively. Phenotypic variation in the biofilm forming ability between strains of the same species (e.g., *Microbacterium lacticum* strain S1 and S2 and *S. rhizophila* S2 and S4 among the dairy strains) was also noticed as shown in Figure 3B. Results of biofilm-forming ability of all strains from the three industries are given in Supplementary material File 1 (sheet 1–3).

### Synergistic interactions in four-species biofilms

**Figure 4:**
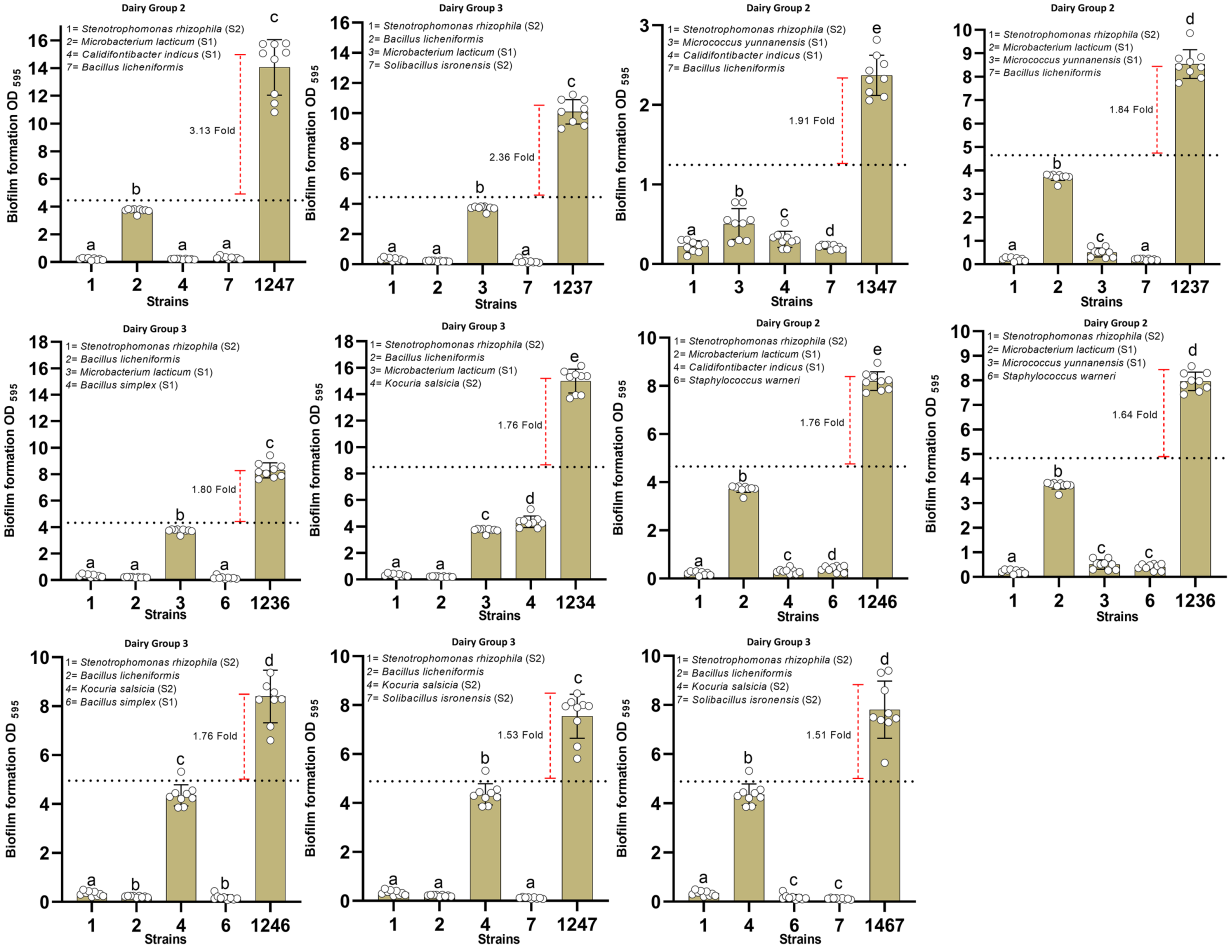
Synergistic interactions in four-species biofilm combinations of dairy strains. All four-species combinations showing pronounced synergistic interactions (at least 1.5-fold increase in biofilm mass compared with sum of biofilm masses of all strains in isolation) are shown. These 11 groups belong to two groups of the dairy strains (2 and 3). In each four-species combination, comparisons were made among the biofilm masses of single species and the mixed-species biofilm using one-way ANOVA with Duncan’s *post hoc* test (*p* < 0.05 for significance).

**Figure 5:**
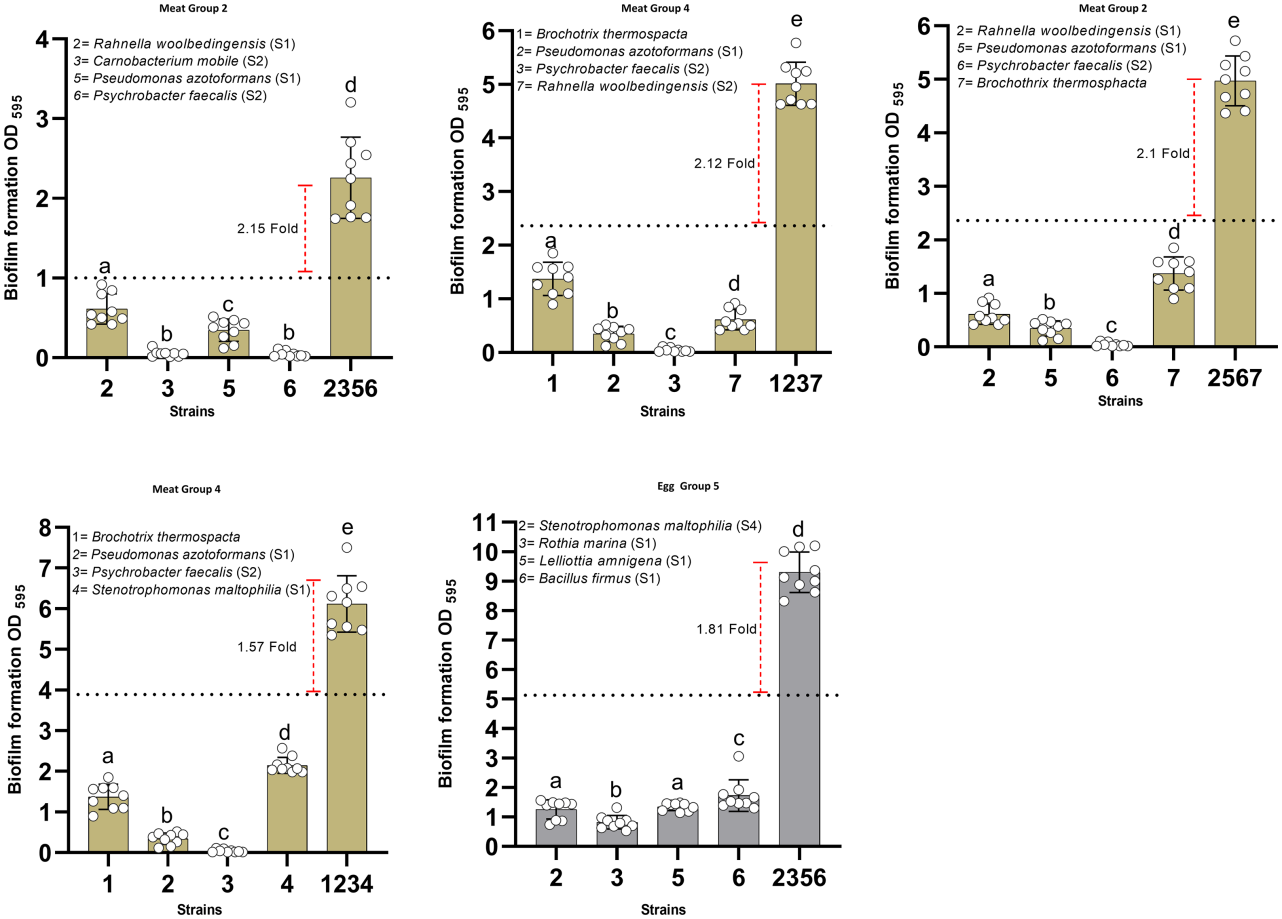
Synergistic interactions in four-species biofilm combinations of meat and egg processing strains. All four-species combinations showing pronounced synergistic interactions (at least 1.5-fold increase in biofilm mass compared with sum of biofilm masses of all strains in isolation) are shown. Only one four-species strain combination from food contact surfaces of egg processing industries showed synergy in biofilms (diagram with the gray bars). In each four-species combination, comparisons were made among the biofilm masses of single species and the mixed-species biofilm using one-way ANOVA with Duncan’s *post hoc* test (*p* < 0.05 for significance).

Bacterial strains from each industry were divided into different groups, each containing 7 different species. This led to the formation of 13 different groups which included 4, 4, and 5 groups of strains belonging to dairy, meat, and egg processing environments, respectively. Bacteria within each group were mixed in all possible combinations of four species and a total of 455 four-species combinations (35 combinations in each group) were developed to find bacterial combinations that show synergy in biofilm mass. About 90% of the four-species combinations had biomass mass lower than the sum of biofilm masses of individual strains within the combination. Eight combinations showed no significant difference (*p* > 0.05) in biofilm mass compared to the sum of biofilm masses of corresponding mono-species biofilms. Only 34 four-species combinations showed synergy as indicated by enhanced biomass production (*p* < 0.05) when compared with sum of biofilm masses of all single-species cultures. Out of these 34 combinations, 16 combinations showed higher than 1.5-fold increase in biofilm mass and interestingly 11 combinations belonged to two groups of dairy strains (Figure 4). Four combinations showing synergy were composed of bacterial strains of the meat processing environment; whereas, only one four-species combination out of 175 four-species combinations comprising strains of the egg processing facility showed synergy where biofilm mass was increased by only 1.57-fold (Figure 5). The presence of *Bacillus cereus* as the fourth species in any combination resulted in reduced biofilm mass that would otherwise be formed with another species in the same combination (sheet 4 and 6–7 in Supplementary Material File 1). For instance, co-culturing of *B. cereus* with three species (*S. rhizophila*, *B. licheniformis*, and *M. lacticum*) resulted in the lowest quantity of biofilm mass compared to the addition of *C. indicus*, *B. simplex*, and *Solibacillus isronensis* as the fourth species (Supplementary Figure S1 derived from groups 3 of dairy strains).

### Specific bacteria involved in synergistic multispecies biofilms

**Figure 6:**
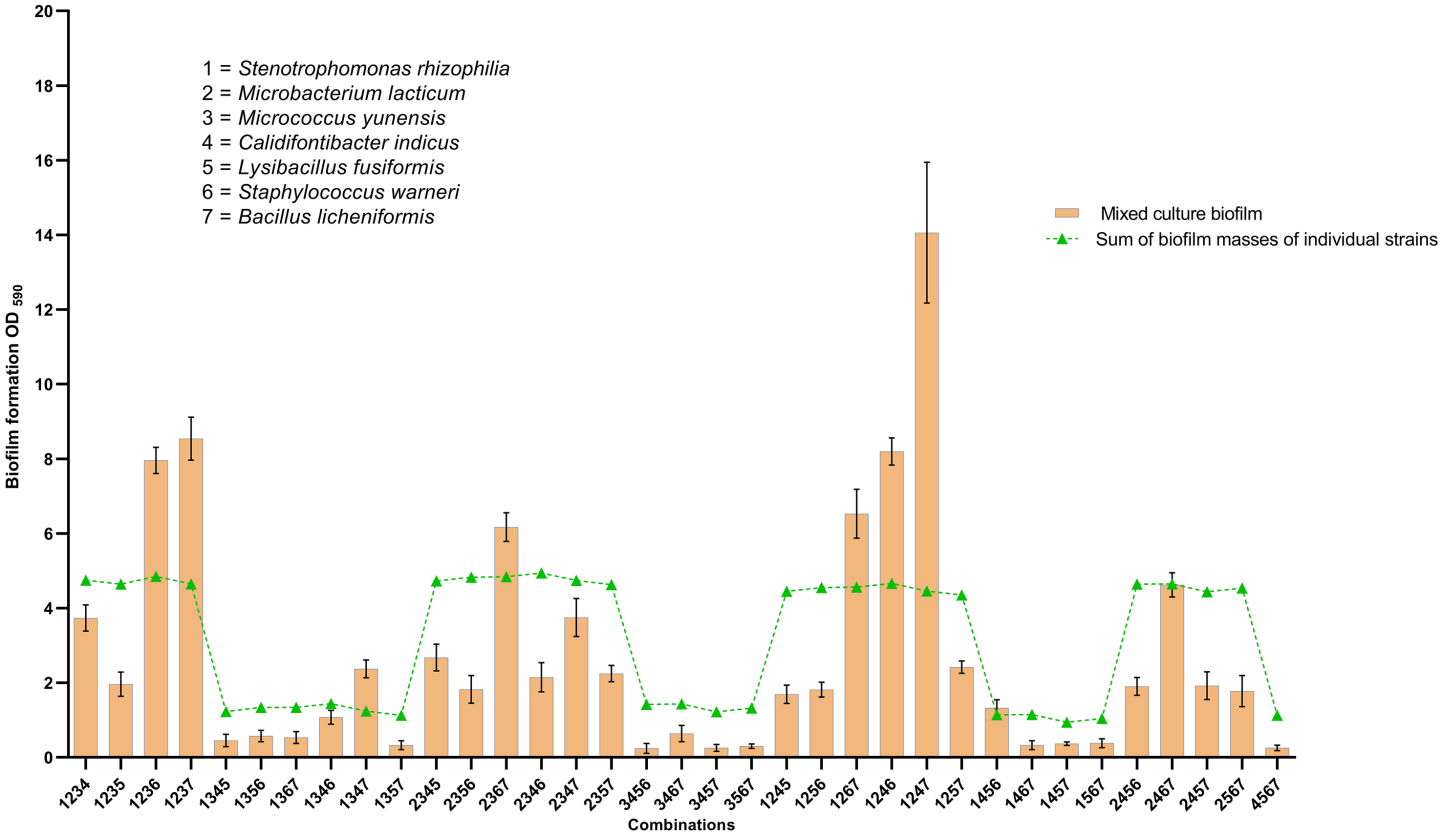
Group No. 2 comprising 7 species isolated from a milk processing industry following cleaning and disinfection. These 7 species were permutated in all possible four-species biofilm combinations (35) to find synergy in four-species biofilms. Out of 35 combinations, 8 combinations turned out to be synergistic in which biofilm mass of the four-species community was higher (*p* < 0.05) than sum of biofilm masses of all four species.

**Figure 7:**
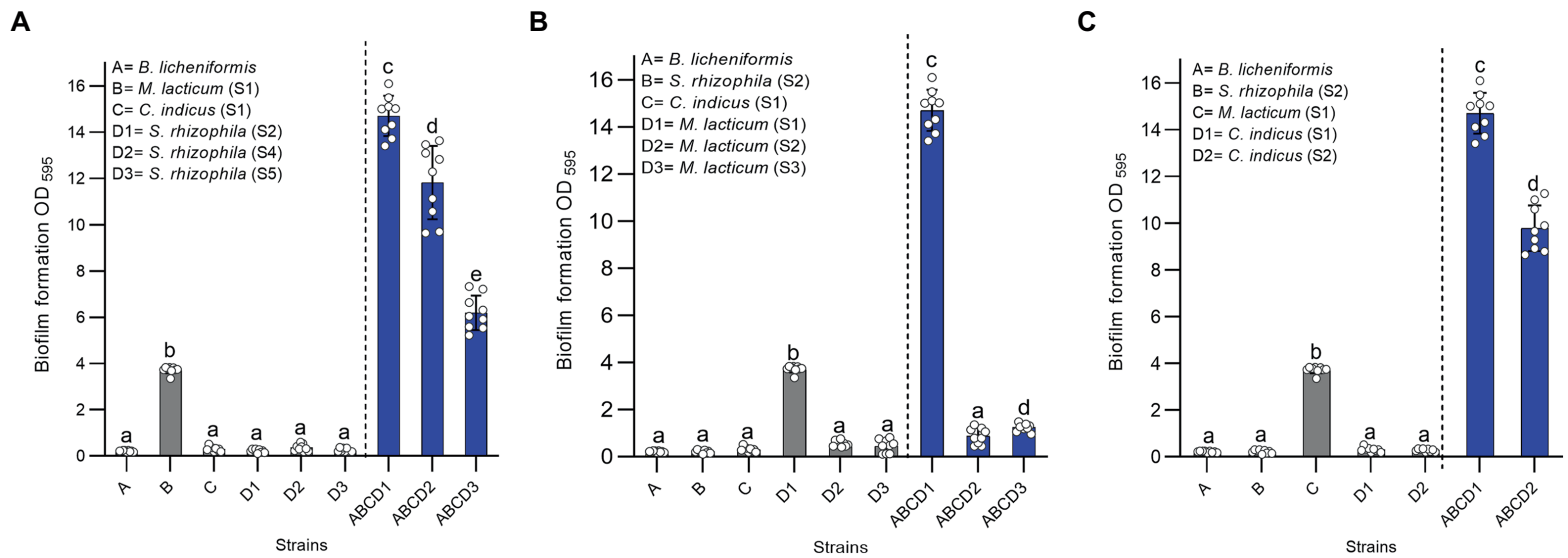
Role of different strains of members of a four-species biofilm community in the overall synergy. **(A)** shows differential influence of three strains of *Stenotrophomonas rhizophila* (S2, S4, and S5) on the synergy observed in the mixed-species biofilm community. **(B)** shows the influence of different strains (S1, S2, and S3) of *Microbacterium lacticum* on the synergy observed in the four-species biofilm. **(C)** shows the influence of two strains (S1, S2) of *Calidifontibacter indicus* on the synergy observed in the four-species biofilm. Lower case letters above bars indicate significant differences among biofilm mass of single and mixed species biofilms that were calculated using one-way ANOVA with Duncan’s *post hoc* test (*p* < 0.05 for significance).

Highest synergy (3.13-fold increase in biofilm mass) in four-species biofilms was observed in combination 1–2–4-7 (*S. rhizophila* (S1), *M. lacticum* (S1), *C. indicus* (S1), and *B. licheniformis*, respectively) of dairy group 2 (Figure 6), where only *M. lacticum* was capable of forming abundant biofilm in mono-species. Interestingly all 11 synergistic biofilm combinations of dairy strains contained *S. rhizophila*, 9 contained *B. licheniformis*, and 7 contained *M. lacticum* (Figure 4). This implies significance of these three species in synergistic biofilms on food contact surfaces in the dairy industry. When strains of *S. rhizophila* (S2), *M. lacticum* (S1), and *C. indicus* (S1) in the combination 1–2–4-7 were replaced, one by one, by other strains of the same species, the observed synergy dramatically changed and turned out to be strain-specific (Figure 7). There was only one strain of *B. licheniformis* in the collection, so strain-specific effect of this species on the overall synergy could not be determined.

**Figure 8:**
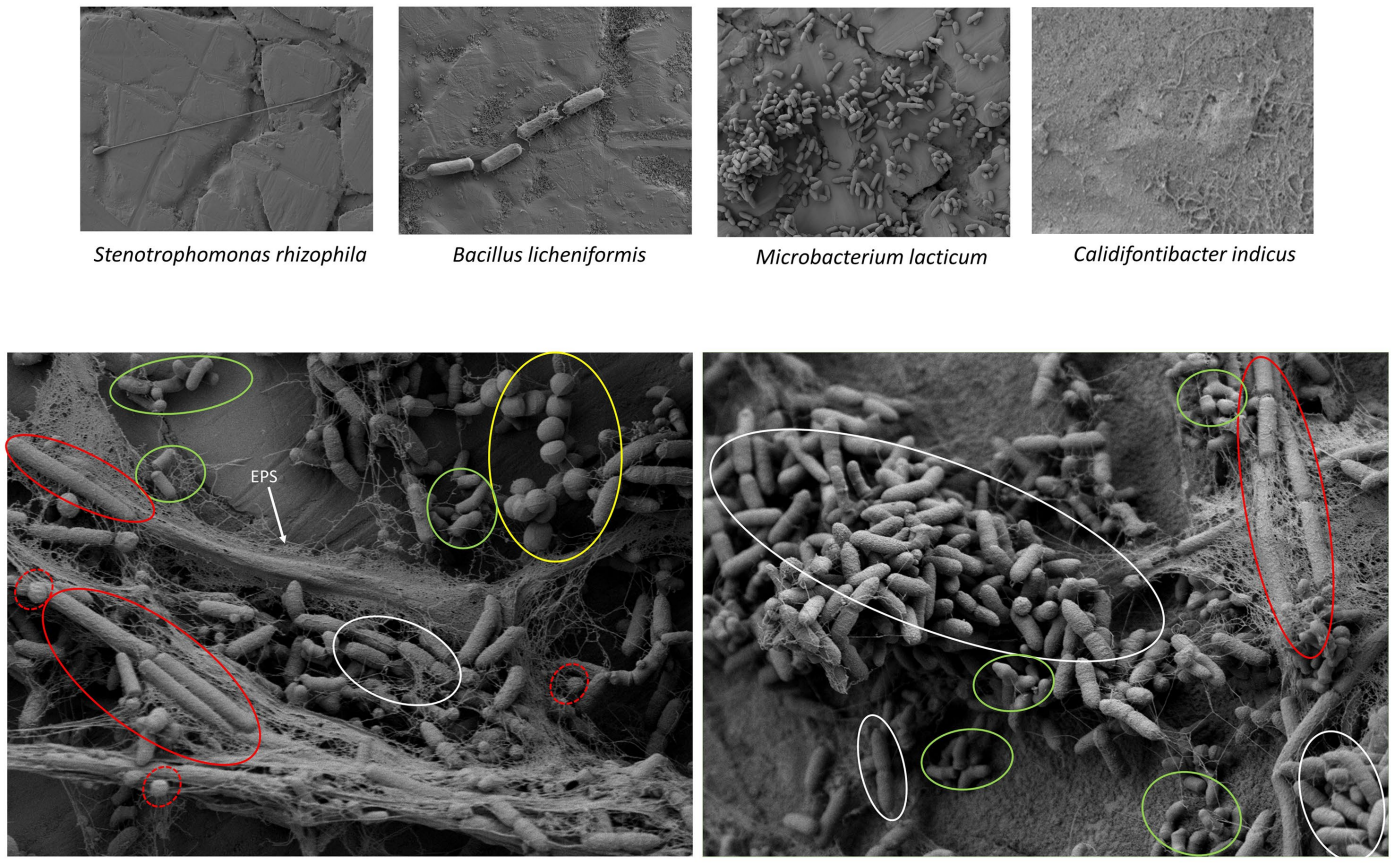
Scanning electron microscopy images of the synergistic biofilm formed by four species of the dairy origin: *Stenotrophomonas rhizophila* (S2), *Microbacterium lacticum* (S1), *Calidifontibacter indicus* (S1), and *Bacillus licheniformis*. The presence of all these four species was confirmed in SEM images and they are highlighted with circles of white, green, yellow, and red colors, respectively. *B. licheniformis* spores are also shown in dotted circle of red color. The first part of the figure shows monospecies biofilms of all species that were present in this selected four-species biofilm combination.

SEM image (Figure 8) of single and the four-species biofilm combination (1–2–4-7) confirms congruence in results related to the biofilm-forming ability of these bacteria on polystyrene and SS. Results of biofilm formation on microtiter plates showed that *S. rhizophila*, *B. licheniformis*, and *C. indicus* are weak biofilm formers and using SEM we could only observe a few cells on the surface for these species, except the image of *C. indicus* where no cells could be seen. Only *M. lacticum* appeared to be a strong biofilm former and SEM images also showed a large number of cells of this species on stainless steel surfaces. SEM images also confirm the presence of all species in the four-species combination – apparently in different proportions – where *S. rhizophila* appears to be dominant.

For the four-species combinations that contained isolates from food contact surfaces of meat processing plants, highest biofilm biomass production was observed in the combination containing *Rahnella woolbedingensis*, *Carnobacterium mobile*, *Pseudomonas azotoformans*, and *Psychrobacter faecalis*. Interestingly, *P. azotoformans* and *P. faecalis* were present in all four combinations; whereas, *R. woolbedingensis* and *Brochothrix thermosphacta* were present in 3/4 four-species combinations that showed synergy (≥ 1.5-fold increase in biofilm mass) (Figure 5). Isolates recovered from food contact surfaces of egg processing industries showed synergy only in one four-species combination that included *S. maltophilia*, *Rothia marina*, *L. amnigena*, and *Bacillus firmus*.

## Discussion

A few studies have explored bacterial diversity on food contact surfaces after routine C&D in an effort to find bacterial contaminants that possibly have a potential role in food contamination and spoilage (Røder et al., 2015; Fagerlund et al., 2017; Bridier et al., 2019; Maes et al., 2019; Wagner et al., 2020). In our previous trial, 87% of the bacterial isolates that were recovered from diverse food processing industries after C&D showed spoilage potential under laboratory conditions (Maes et al., 2019). Biofilm-forming ability of bacterial contaminants is intrinsically linked to their ability to adapt to conditions on food processing surfaces or to interact with other bacteria within food processing facilities and their resistance against antimicrobials (e.g., C&D) (Sadiq et al., 2017a; Yuan et al., 2020). The focus of the current study was to determine the biofilm-forming potential of some of those isolates and identify synergistic interactions among the isolates in multispecies biofilm combinations. Similar approaches to monitor synergy among bacterial four-species biofilms have been used on bacteria recovered from soil (Ren et al., 2015) or human fecal samples (Sadiq et al., 2021b).

The use of bacterial strains collected at the same time from the same food contact surfaces, rather than using laboratory collections of model microorganisms is of importance when interactions in mixed species biofilms are to be assessed because of differences in bacterial strains. Many previous studies have also reported the biofilm-forming ability of many isolates recovered from dairy (Cherif-Antar et al., 2016; Sadiq et al., 2017b) and meat (Stanford et al., 2021; Wagner et al., 2021) processing industries. Different food processing surfaces have unique microbial profiles influenced by the processing environment, processing techniques, and type and quality of raw materials. Thus, the outcomes of many trials mapping microbial abundance and determining the biofilm-forming capacities of recovered bacterial isolates provide new knowledge and confirm the prevalence of most problematic species or common key microbial contaminants of food industries in different parts of the world (Maes et al., 2019; Wagner et al., 2020, 2021). The ability of many bacteria to form biofilms is influenced by other species on surfaces due to many ecological interactions. A number of recent studies cataloging bacterial diversity on food contact surfaces and studying bacterial interspecies interactions have concluded that biofilms in the food industry are often comprised of multiple species that interact with each other mainly due to metabolic and physiological needs (Wagner et al., 2021; Nan et al., 2022; Voglauer et al., 2022). In our trial, 35% of the isolates turned out to be strong biofilm formers which shows that many species probably co-existed on surfaces with other resident microbiota as a result of positive or negative associations. In a positive interaction, one species promotes growth of another species usually through nutrient availability and by creating new niches, whereas in a negative interaction species inhibit growth of other species through nutrient exploitation or by producing antagonistic chemicals (Kehe et al., 2021).

Many recent studies have shown how the presence of certain bacterial species in a community profoundly affects the biofilm-forming potential or often growth of other species either through cooperative or competitive interactions. For instance, the presence of antagonistic substances produced by dairy strains of *B. cereus* were shown to negatively affect the growth of *Listeria monocytogenes* in dual-species biofilms (Alonso et al., 2020). *Bacillus safensis*, from a meat processing plant, was reported to inhibit the growth of *L. monocytogenes* by 4 log CFU/cm^2^ in a dual species biofilm comprising *L. monocytogenes* and *Salmonella enterica* (Ripolles-Avila et al., 2022). *Pseudomonas putida*, a resident bacterial species in drinking water systems of broiler houses, was reported to inhibit growth and biofilm formation by *Salmonella* Java in mixed culture (Maes et al., 2020). On the other hand, many species induce biofilm formation in other species by producing signaling molecules like H_2_O_2_ (Duan et al., 2016), autoinducer 2 (AI-2) (Laganenka and Sourjik, 2018) or through physical contact (Xu et al., 2017).

### Prevalence of negative interactions in four-species biofilms

About 90% of our four-species biofilm interactions of turned out to be negative or neutral (1.8%) which is not surprising. Contrary to this, a similar trial on 7 soil isolates that were randomly mixed into 35 four-species combinations showed synergy in 63% combinations (Ren et al., 2015). The use of a limited number of species (7) and a different definition of synergy where combined biofilm mass was compared with the biofilm mass of the single best biofilm former renders comparison of the two studies difficult.

There is a lot of controversy about the importance of negative interactions in microbial communities. One of the opinions is bacterial species rarely work together and negative interactions prevail in microbial communities, and cooperation, where two strains both benefit, is typically rare. Foster and Bell (2012) studied fitness interactions among bacterial strains, collected from water-filled tree holes, in a randomly chosen pairwise mixtures as well as higher-order interactions, by comparing the productivity of single-species and mixed-species cultures of 72 bacterial species in planktonic cultures and concluded that the majority of pairwise species combinations were net negative. In addition, net positive interactions in higher-order interactions were rare too. On the other hand, a study on 180,408 pair-wise interactions, under 40 carbon sources, among 20 different bacteria of soil origin reported 24% interactions being cooperative in which the total coculture yield was higher than the sum of yields from monoculture, and these interactions depended on strain pair and carbon source. It was concluded that positive interactions are highly abundant, but the conditions under which they emerge are difficult to predict (Kehe et al., 2021). Bacteria opt to compete in nutrient-rich conditions and cooperate in nutrient-limited conditions (Hoek et al., 2016), thus the use of BHI (a highly nutrient liquid medium) in four-species biofilm forming trials may have led to competitive interactions.

In our trial, positive interactions (synergy in biofilms) were focused as these growth-promoting interactions greatly affect the productivity and diversity of natural and industrial microbial communities (Burmølle et al., 2006; Ren et al., 2015). Competitive interactions on the one hand are vital for the maintenance of ecological diversity and evolution, and on the other hand, they act as an ecological force that play an important role in microbial metabolism and ecological and evolutionary diversity (Day and Young, 2004). Any net negative interaction seen in a laboratory four-species co-cultivation model can possibly be equilibrated into a competitive balance in a natural setting where numerous other species coexist (Kehe et al., 2021; Lyng and Kovács, 2023). Nevertheless, natural interactions are often far more complex than cultivations in the laboratory so sometimes it is difficult to predict bacterial behavior in a natural setting from laboratory trials.

Most of the biofilm forming strains belonged to the egg processing industry, whereas least synergy was observed among its four-species biofilm combinations and the opposite was the case for the dairy strains. One explanation is egg isolates were collected from diverse sampling points, whereas most of the bacterial contaminants of the dairy industry after C&D were originated from the surface of pasteurizers (Maes et al., 2019).

### Synergy in specific biofilm combinations of strains isolated from the dairy industry

Among the dairy strains, highest synergy was found among all combinations containing *S. rhizophila*, *B. licheniformis* and *M. lacticum*. These three species are known as problematic species in the dairy industry due to their spoilage potential and prevalence. *Stenotrophomonas* species are one of the widely reported bacterial contaminants in milk and dairy products (Boubendir et al., 2016; Zeinhom et al., 2021). *B. licheniformis* is one of the biggest concerns for the dairy industry throughout the world, especially for milk powder production (Sadiq et al., 2016), as it is the predominant bacterial species found in raw milk and at all stages of dairy processing (Banykó and Vyletělová, 2009; Gopal et al., 2015; Sadiq et al., 2016, 2018). *B. licheniformis* and *M. lacticum* have been reported co-existing in whey protein concentrate (Walsh et al., 2012) and raw milk (Ribeiro-Júnior et al., 2020). These three species were shown to form synergistic biofilms in many combinations with other species like *B. simplex*, *Kocuria salsicia*, and opportunistic pathogens like *Staphylococcus warneri*. Interestingly, all these species have been reported to co-exist in biofilm samples of milking machines in dairy farms too (Weber et al., 2019). SEM images show that after 24 h no exclusion of species took place and all species appeared to form a stable community. Apparent dominance of *S. rhizophila* and *B. licheniformis* show that these species not only form biofilms, but dominate in the community when co-existing with other species. In addition, EPS formation for these strains seems to be a community-specific characteristic as no EPS was seen in any of the mono-species biofilms, however from these findings it remained unclear which species produced EPS in the community. The strain-specific nature of synergy in mixed-species biofilms on the one hand emphasizes the significance of strains, rather than species, in studying bacterial interactions and on the one hand, it has implications for the development of synthetic bacterial communities. The role of inter-strain phenotypic diversity in modulating interactions in mixed species biofilms has been reported, mainly in dual-species biofilm settings (Gomes-Fernandes et al., 2022). *L. monocytogenes* strains of food origin have been reported to show large variations in competitive growth in different mixed-species biofilm settings (Heir et al., 2018). Our findings show that synergistic interactions in multispecies biofilms are strain-dependent.

The observed antagonistic behavior of *B. cereus* may be related to the production of inhibitory compounds by *B. cereus* as previously reported (Alonso et al., 2020). The prevalence of competition due to the presence of certain species gives hope for potential strategies related to bacterial community engineering that seek to expel or kill certain species without the need for antimicrobials. This approach of community coalescence can be used to identify potentially “facilitating” and “inhibiting” strains in different environmental settings.

### Synergy in specific biofilm combinations of strains isolated from the meat and egg industries

When examining the multispecies biofilms formed by meat processing isolates, certain isolates (e.g., *P. azotoformans* and *P. faecalis*) appeared to promote synergistic biofilm formation more frequently than others as they were a part of all four-species biofilm combinations showing higher synergies. These species appeared to form synergistic biofilms with other species like *S. maltophilia* and *Br. thermosphacta*. Interestingly, *P. azotoformans* and *P. faecalis* are also among the bacterial strains which are considered as important contaminants affecting the quality of meat products throughout the world (Røder et al., 2015; Circella et al., 2020). *Br. thermosphacta* was present in 3 out of 4 four-species biofilm combinations indicating its importance in the observed synergy. This species is one of main microbial quality issues and associated with spoilage of meat and seafood (Nowak et al., 2012; Patange et al., 2017; Gaillac et al., 2022). Duthoo et al. (2021) reported highly diverse bacteria on the surface of chicken and ham slicer in a meat processing industry; interestingly, 70% of those isolates were later recovered from cooked chicken and ham products too and included *Br. thermosphacta*, *Carnobacterium* and *Psychrobacter* species, among others. *Pseudomonas* sp. (> 50%), *Psychrobacter* sp. and *Br. thermosphacta* were also found to be the dominant bacterial communities associated with the spoilage of beef (De Filippis et al., 2013). Wagner et al. (2020) evaluated 47 food contact and 61 non-food contact surfaces, during processing and after C&D, within a meat processing industry in Austria and reported that the most prevalent bacteria belonged to the genera *Brochothrix* (present in 80% of biofilms), *Psychrobacter* (present in 70% of biofilms) and *Pseudomonas*.

Only one four-species combination among the isolates from an egg processing industry appeared to have synergy despite a large diversity of bacteria present on egg processing surfaces following C&D. This indicates that bacterial co-existence does not necessarily require synergistic interactions. Species facing competition in microbial communities might evolve diverse mechanisms that promote coexistence and do not necessarily lead to exclusion (Hibbing et al., 2010).

## Concluding remarks

Data generated from this trial provide insights into the ability of co-localized bacterial isolates from diverse food industries to form synergistic biofilms with high relevance for food production facilities. Our findings indicate that the capability of a bacterial strain to engage in a biofilm community and thereby persist in processing facilities cannot be assessed merely based on its capability of monoculture surface attachment and biofilm formation. Some simplified experimental models of interacting bacterial communities are reported in this work that likely co-exist in biofilms on food contact surfaces and survive C&D regimes as a result of some unknown interactions. Interestingly, almost all bacterial species in the reported synergistic four-species combinations are widely recognized as key problematic species in respective food industries. The generated knowledge on species’ co-occurrence can be used by ecologists to discern the forces that dictate community structure on food contact surfaces in different industries. Our work has demonstrated strain-specific synergistic interactions in multispecies biofilms recovered from food contact surfaces. This implies that strain variations play a role in the modulation of microbial community dynamics which have implications for the development of biofilm control strategies.

The nature of underlying interactions (i.e., metabolic dependencies or physical interactions) among these bacterial species remains unknown; however, the role of these interactions in bacterial resilience on surfaces and antimicrobial tolerance is highly anticipated. A number of different interspecies ecological interactions – cooperative (mutually beneficial) to antagonistic (detrimental to one or both species) – within each four-species biofilm community may be involved in governing the fate of individual entities in the community. Understanding of these interactions is vital to develop strategies to prevent and reduce bacterial biofilms in the food production facility.

## Data availability statement

The original contributions presented in the study are included in the article/Supplementary material, further inquiries can be directed to the corresponding authors.

## Author contributions

FS is the recipient of the project grant and he carried out the experiments and wrote the draft. The part of the work related to culture collection and identification of strains was done by SM. KR, MB, and MH supervised the project and revised the manuscript. All authors contributed to the article and approved the submitted version.

## Funding

This work was funded under EXCELLENT SCIENCE - Marie Skłodowska-Curie Actions – a program of the European Commission (Grant agreement ID: 101025683). We also acknowledge Villum Foundation, project number 35906 for financial support given to Mette Burmølle. The funding Research Executive Agency (REA), delegated by the European Commission, is not responsible for any use that may be made of the information this article contains.

## Conflict of interest

The authors declare that the research was conducted in the absence of any commercial or financial relationships that could be construed as a potential conflict of interest.

## Publisher’s note

All claims expressed in this article are solely those of the authors and do not necessarily represent those of their affiliated organizations, or those of the publisher, the editors and the reviewers. Any product that may be evaluated in this article, or claim that may be made by its manufacturer, is not guaranteed or endorsed by the publisher.

## Supplementary material

The Supplementary material for this article can be found online at: https://www.frontiersin.org/articles/10.3389/fmicb.2023.1159434/full#supplementary-material

## References

[ref1] Adamowicz E. M. Flynn J. Hunter R. C. Harcombe W. R. (2018). Cross-feeding modulates antibiotic tolerance in bacterial communities. ISME J. 12, 2723–2735. doi: 10.1038/s41396-018-0212-z, PMID: 29991761 PMC6194032

[ref2] Alonso V. P. P. Harada A. M. M. Kabuki D. Y. (2020). Competitive and/or cooperative interactions of listeria monocytogenes with Bacillus cereus in dual-species biofilm formation. Front. Microbiol. 11, doi: 10.3389/fmicb.2020.00177 PMC7058548 32184763

[ref3] Banykó J. Vyletělová M. (2009). Determining the source of Bacillus cereus and bacillus licheniformis isolated from raw milk, pasteurized milk and yoghurt. Lett. Appl. Microbiol. 48, 318–323. doi: 10.1111/j.1472-765X.2008.02526.x, PMID: 19187503

[ref4] Bottery M. J. Matthews J. L. Wood A. J. Johansen H. K. Pitchford J. W. Friman V.-P. (2022). Inter-species interactions alter antibiotic efficacy in bacterial communities. ISME J. 16, 812–821. doi: 10.1038/s41396-021-01130-6, PMID: 34628478 PMC8857223

[ref5] Boubendir A. Serrazanetti D. I. Hamidechi M. A. Vannini L. Guerzoni M. E. (2016). Changes in bacterial populations in refrigerated raw milk collected from a semi-arid area of Algeria. Ann. Microbiol. 66, 777–783. doi: 10.1007/s13213-015-1163-5

[ref6] Bridier A. Le Grandois P. Moreau M.-H. Prénom C. Le Roux A. Feurer C.. (2019). Impact of cleaning and disinfection procedures on microbial ecology and salmonella antimicrobial resistance in a pig slaughterhouse. Sci. Rep. 9:12947. doi: 10.1038/s41598-019-49464-8, PMID: 31506516 PMC6736965

[ref7] Burmølle M. Webb J. S. Rao D. Hansen L. H. Sørensen S. J. Kjelleberg S. (2006). Enhanced biofilm formation and increased resistance to antimicrobial agents and bacterial invasion are caused by synergistic interactions in multispecies biofilms. Appl. Environ. Microbiol. 72, 3916–3923. doi: 10.1128/AEM.03022-05, PMID: 16751497 PMC1489630

[ref8] Cherif-Antar A. Moussa Boudjemâa B. Didouh N. Medjahdi K. Mayo B. Flórez A. B. (2016). Diversity and biofilm-forming capability of bacteria recovered from stainless steel pipes of a milk-processing dairy plant. Dairy Sci. Technol. 96, 27–38. doi: 10.1007/s13594-015-0235-4

[ref9] Circella E. Schiavone A. Barrasso R. Camarda A. Pugliese N. Bozzo G. (2020). Pseudomonas azotoformans belonging to Pseudomonas fluorescens group as causative agent of blue coloration in carcasses of slaughterhouse rabbits. Animals (Basel) 10. doi: 10.3390/ani10020256, PMID: 32041142 PMC7070765

[ref10] Coughlan L. M. Cotter P. D. Hill C. Alvarez-Ordóñez A. (2016). New weapons to fight old enemies: novel strategies for the (bio)control of bacterial iofilms in the food industry. Front. Microbiol. 7:e01641. doi: 10.3389/fmicb.2016.01641, PMID: 27803696 PMC5067414

[ref11] Day T. Young K. A. (2004). Competitive and facilitative evolutionary diversification. Bioscience 54, 101–109. doi: 10.1641/0006-3568(2004)054[0101:CAFED]2.0.CO;2, PMID: 36711513

[ref12] De Filippis F. La Storia A. Villani F. Ercolini D. (2013). Exploring the sources of bacterial spoilers in beefsteaks by culture-independent high-throughput sequencing. PLoS One 8:e70222. doi: 10.1371/journal.pone.0070222, PMID: 23936168 PMC3723795

[ref13] Duan D. Scoffield J. A. Zhou X. Wu H. (2016). Fine-tuned production of hydrogen peroxide promotes biofilm formation of streptococcus parasanguinis by a pathogenic cohabitant Aggregatibacter actinomycetemcomitans. Environ. Microbiol. 18, 4023–4036. doi: 10.1111/1462-2920.13425, PMID: 27348605 PMC5118171

[ref14] Duthoo E. Rasschaert G. Leroy F. Weckx S. Heyndrickx M. De Reu K. (2021). The microbiota of modified-atmosphere-packaged cooked charcuterie products throughout their shelf-life period, as revealed by a complementary combination of culture-dependent and culture-ondependent analysis. Microorganisms 9. doi: 10.3390/microorganisms9061223, PMID: 34200022 PMC8229102

[ref15] Fagerlund A. Langsrud S. Møretrø T. (2021). Microbial diversity and ecology of biofilms in food industry environments associated with listeria monocytogenes persistence. Curr. Opin. Food Sci. 37, 171–178. doi: 10.1016/j.cofs.2020.10.015

[ref16] Fagerlund A. Møretrø T. Heir E. Briandet R. Langsrud S. (2017). Cleaning and disinfection of biofilms composed of listeria monocytogenes and background microbiota from meat processing surfaces. Appl. Environ. Microbiol. 83, e01046–e01017. doi: 10.1128/AEM.01046-17 28667108 PMC5561291

[ref17] Foster K. Bell T. (2012). Competition, not cooperation, dominates interactions among culturable microbial species. Curr. Biol. 22, 1845–1850. doi: 10.1016/j.cub.2012.08.005, PMID: 22959348

[ref18] Gaillac A. Briandet R. Delahaye E. Deschamps J. Vigneau E. Courcoux P.. (2022). Exploring the diversity of biofilm formation by the food spoiler Brochothrix thermosphacta. Microorganisms 10:2474. doi: 10.3390/microorganisms10122474, PMID: 36557727 PMC9785830

[ref19] Goh Y. F. Røder H. L. Chan S. H. Ismail M. H. Madsen J. S. Lee K. W. K.. (2023). Associational resistance to predation by protists in a mixed species biofilm. Appl. Environ. Microbiol. 89, e01741–e01722. doi: 10.1128/aem.01741-22 36656007 PMC9972941

[ref20] Gomes-Fernandes M. Gomez A.-C. Bravo M. Huedo P. Coves X. Prat-Aymerich C.. (2022). Strain-specific interspecies interactions between co-isolated pairs of Staphylococcus aureus and Pseudomonas aeruginosa from patients with tracheobronchitis or bronchial colonization. Sci. Rep. 12:3374. doi: 10.1038/s41598-022-07018-5, PMID: 35233050 PMC8888623

[ref21] Gopal N. Hill C. Ross P. R. Beresford T. P. Fenelon M. A. Cotter P. D. (2015). The prevalence and control of bacillus and related spore-forming bacteria in the dairy industry. Front. Microbiol. 6:e01418. doi: 10.3389/fmicb.2015.01418, PMID: 26733963 PMC4685140

[ref22] Heir E. Møretrø T. Simensen A. Langsrud S. (2018). Listeria monocytogenes strains show large variations in competitive growth in mixed culture biofilms and suspensions with bacteria from food processing environments. Int. J. Food Microbiol. 275, 46–55. doi: 10.1016/j.ijfoodmicro.2018.03.026, PMID: 29631210

[ref23] Hibbing M. E. Fuqua C. Parsek M. R. Peterson S. B. (2010). Bacterial competition: surviving and thriving in the microbial jungle. Nat. Rev. Microbiol. 8, 15–25. doi: 10.1038/nrmicro2259, PMID: 19946288 PMC2879262

[ref24] Highmore C. J. Melaugh G. Morris R. J. Parker J. Direito S. O. L. Romero M.. (2022). Translational challenges and opportunities in biofilm science: a BRIEF for the future. NPJ Biofilm. Microb. 8:68. doi: 10.1038/s41522-022-00327-7, PMID: 36038607 PMC9424220

[ref25] Hoek T. A. Axelrod K. Biancalani T. Yurtsev E. A. Liu J. Gore J. (2016). Resource availability modulates the cooperative and competitive nature of a microbial cross-feeding mutualism. PLoS Biol. 14:e1002540. doi: 10.1371/journal.pbio.1002540, PMID: 27557335 PMC4996419

[ref26] Karki A. B. Ballard K. Harper C. Sheaff R. J. Fakhr M. K. (2021). Staphylococcus aureus enhances biofilm formation, aerotolerance, and survival of campylobacter strains isolated from retail meats. Sci. Rep. 11:13837. doi: 10.1038/s41598-021-91743-w, PMID: 34226590 PMC8257638

[ref27] Karygianni L. Ren Z. Koo H. Thurnheer T. (2020). Biofilm matrixome: extracellular components in structured microbial communities. Trends Microbiol. 28, 668–681. doi: 10.1016/j.tim.2020.03.016, PMID: 32663461

[ref28] Kehe J. Ortiz A. Kulesa A. Gore J. Blainey P. C. Friedman J. (2021). Positive interactions are common among culturable bacteria. Sci. Adv. 7:eabi7159. doi: 10.1126/sciadv.abi7159 34739314 PMC8570599

[ref29] Kommerein N. Doll K. Stumpp N. S. Stiesch M. (2018). Development and characterization of an oral multispecies biofilm implant flow chamber model. PLoS One 13:e0196967. doi: 10.1371/journal.pone.0196967, PMID: 29771975 PMC5957423

[ref30] Laganenka L. Sourjik V. (2018). Autoinducer 2-dependent Escherichia coli biofilm formation is enhanced in a dual-species coculture. Appl. Environ. Microbiol. 84, e02638–e02617. doi: 10.1128/AEM.02638-17 29269492 PMC5812939

[ref31] Langsrud S. Moen B. Møretrø T. Løype M. Heir E. (2016). Microbial dynamics in mixed culture biofilms of bacteria surviving sanitation of conveyor belts in salmon-processing plants. J. Appl. Microbiol. 120, 366–378. doi: 10.1111/jam.13013, PMID: 26613979

[ref32] Liu W. Russel J. Røder H. L. Madsen J. S. Burmølle M. Sørensen S. J. (2017). Low-abundant species facilitates specific spatial organization that promotes multispecies biofilm formation. Environ. Microbiol. 19, 2893–2905. doi: 10.1111/1462-2920.13816, PMID: 28618083

[ref33] Lyng M. Kovács Á. T. (2023). Frenemies of the soil: bacillus and pseudomonas interspecies in teractions. Trends Microbiol. doi: 10.1016/j.tim.2023.02.003, PMID: 36878770

[ref34] Maes S. De Reu K. Van Weyenberg S. Lories B. Heyndrickx M. Steenackers H. (2020). Pseudomonas putida as a potential biocontrol agent against salmonella Java biofilm formation in the drinking water system of broiler houses. BMC Microbiol. 20:373. doi: 10.1186/s12866-020-02046-5, PMID: 33308162 PMC7731557

[ref35] Maes S. Heyndrickx M. Vackier T. Steenackers H. Verplaetse A. Reu K. (2019). Identification and spoilage potential of the remaining dominant microbiota on food contact surfaces after cleaning and disinfection in different food industries. J. Food Prot. 82, 262–275. doi: 10.4315/0362-028X.JFP-18-226, PMID: 30682263

[ref36] Mevo S. I. U. Ashrafudoulla M. Furkanur Rahaman Mizan M. Park S. H. Ha S.-D. (2021). Promising strategies to control persistent enemies: some new technologies to combat biofilm in the food industry—a review. Compr. Rev. Food Sci. Food Saf. 20, 5938–5964. doi: 10.1111/1541-4337.12852, PMID: 34626152

[ref37] Nadell C. D. Drescher K. Foster K. R. (2016). Spatial structure, cooperation and competition in biofilms. Nat. Rev. Microbiol. 14, 589–600. doi: 10.1038/nrmicro.2016.84, PMID: 27452230

[ref38] Nan Y. Rodas-Gonzalez A. Stanford K. Nadon C. Yang X. Mcallister T.. (2022). Formation and transfer of multi-species biofilms containing *E. coli* O103:H2 on food contact surfaces to beef. Front. Microbiol. 13:863778. doi: 10.3389/fmicb.2022.994033, PMID: 35711784 PMC9196126

[ref39] Nowak A. Rygala A. Oltuszak-Walczak E. Walczak P. (2012). The prevalence and some metabolic traits of Brochothrix thermosphacta in meat and meat products packaged in different ways. J. Sci. Food Agric. 92, 1304–1310. doi: 10.1002/jsfa.4701, PMID: 22083437

[ref40] Parijs I. Steenackers H. P. (2018). Competitive inter-species interactions underlie the increased antimicrobial tolerance in multispecies brewery biofilms. ISME J. 12, 2061–2075. doi: 10.1038/s41396-018-0146-5, PMID: 29858577 PMC6052168

[ref41] Patange A. Boehm D. Bueno-Ferrer C. Cullen P. J. Bourke P. (2017). Controlling Brochothrix thermosphacta as a spoilage risk using in-package atmospheric cold plasma. Food Microbiol. 66, 48–54. doi: 10.1016/j.fm.2017.04.002, PMID: 28576372

[ref42] Ren D. Madsen J. S. Sørensen S. J. Burmølle M. (2015). High prevalence of biofilm synergy among bacterial soil isolates in cocultures indicates bacterial interspecific cooperation. ISME J. 9, 81–89. doi: 10.1038/ismej.2014.96, PMID: 24936766 PMC4274433

[ref43] Ribeiro-Júnior J. C. Tamanini R. Alfieri A. A. Beloti V. (2020). Effect of milk bactofugation on the counts and diversity of thermoduric bacteria. J. Dairy Sci. 103, 8782–8790. doi: 10.3168/jds.2020-18591, PMID: 32828509

[ref44] Ripolles-Avila C. Guitan-Santamaria M. Pizarro-Giménez K. Mazaheri T. Rodríguez-Jerez J. J. (2022). Dual-species biofilms formation between dominant microbiota isolated from a meat processing industry with listeria monocytogenes and salmonella enterica: unraveling their ecological interactions. Food Microbiol. 105:104026. doi: 10.1016/j.fm.2022.104026, PMID: 35473979

[ref45] Røder H. L. Raghupathi P. K. Herschend J. Brejnrod A. Knøchel S. Sørensen S. J.. (2015). Interspecies interactions result in enhanced biofilm formation by co-cultures of bacteria isolated from a food processing environment. Food Microbiol. 51, 18–24. doi: 10.1016/j.fm.2015.04.008, PMID: 26187823

[ref46] Sadiq F. A. Burmølle M. Heyndrickx M. Flint S. Lu W. Chen W.. (2021a). Community-wide changes reflecting bacterial interspecific interactions in multispecies biofilms. Crit. Rev. Microbiol. 47, 338–358. doi: 10.1080/1040841X.2021.1887079, PMID: 33651958

[ref47] Sadiq F. A. Flint S. He G. (2018). Microbiota of milk powders and the heat resistance and spoilage potential of aerobic spore-forming bacteria. Int. Dairy J. 85, 159–168. doi: 10.1016/j.idairyj.2018.06.003 27657656

[ref48] Sadiq F. A. Flint S. Li Y. Ou K. Yuan L. He G. Q. (2017a). Phenotypic and genetic heterogeneity within biofilms with particular emphasis on persistence and antimicrobial tolerance. Future Microbiol. 12, 1087–1107. doi: 10.2217/fmb-2017-0042, PMID: 28783379

[ref49] Sadiq F. A. Flint S. Yuan L. Li Y. Liu T. He G. (2017b). Propensity for biofilm formation by aerobic mesophilic and thermophilic spore forming bacteria isolated from Chinese milk powders. Int. J. Food Microbiol. 262, 89–98. doi: 10.1016/j.ijfoodmicro.2017.09.015, PMID: 28968534

[ref50] Sadiq F. A. Li Y. Liu T. Flint S. Zhang G. He G. (2016). A RAPD based study revealing a previously unreported wide range of mesophilic and thermophilic spore formers associated with milk powders in China. Int. J. Food Microbiol. 217, 200–208. doi: 10.1016/j.ijfoodmicro.2015.10.030, PMID: 26555161

[ref51] Sadiq F. A. Wenwei L. Heyndrickx M. Flint S. Wei C. Jianxin Z.. (2021b). Synergistic interactions prevail in multispecies biofilms formed by the human gut microbiota on mucin. FEMS Microbiol. Ecol. 97. doi: 10.1093/femsec/fiab096, PMID: 34190973

[ref52] Stanford K. Tran F. Zhang P. Yang X. (2021). Biofilm-forming capacity of Escherichia coli isolated from cattle and beef packing plants: relation to virulence attributes, stage of processing, antimicrobial interventions, and heat tolerance. Appl. Environ. Microbiol. 87:e0112621. doi: 10.1128/AEM.01126-21, PMID: 34550756 PMC8579979

[ref53] Teh K. H. Flint S. Palmer J. Andrewes P. Bremer P. Lindsay D. (2014). Biofilm − an unrecognised source of spoilage enzymes in dairy products? Int. Dairy J. 34, 32–40. doi: 10.1016/j.idairyj.2013.07.002

[ref54] Voglauer E. M. Zwirzitz B. Thalguter S. Selberherr E. Wagner M. Rychli K. (2022). Biofilms in water hoses of a meat processing environment harbor complex microbial communities. Front. Microbiol. 13:e832213. doi: 10.3389/fmicb.2022.832213, PMID: 35237250 PMC8882869

[ref55] Wagner E. M. Fischel K. Rammer N. Beer C. Palmetzhofer A. L. Conrady B.. (2021). Bacteria of eleven different species isolated from biofilms in a meat processing environment have diverse biofilm forming abilities. Int. J. Food Microbiol. 349:109232. doi: 10.1016/j.ijfoodmicro.2021.109232, PMID: 34022615

[ref56] Wagner E. M. Pracser N. Thalguter S. Fischel K. Rammer N. Pospíšilová L.. (2020). Identification of biofilm hotspots in a meat processing environment: detection of spoilage bacteria in multi-species biofilms. Int. J. Food Microbiol. 328:108668. doi: 10.1016/j.ijfoodmicro.2020.108668, PMID: 32474228

[ref57] Walsh C. Meade J. Mcgill K. Fanning S. (2012). The biodiversity of thermoduric bacteria isolated from whey. J. Food Saf. 32, 255–261. doi: 10.1111/j.1745-4565.2012.00375.x

[ref58] Wang N. Jin Y. He G. Yuan L. (2021). Development of multi-species biofilm formed by thermophilic bacteria on stainless steel immerged in skimmed milk. Food Res. Int. 150:110754. doi: 10.1016/j.foodres.2021.110754, PMID: 34865772

[ref59] Wang C. Van Der Mei H. C. Busscher H. J. Ren Y. (2020). Streptococcus mutans adhesion force sensing in multi-species oral biofilms. NPJ Biofilm Microb. 6:25. doi: 10.1038/s41522-020-0135-0, PMID: 32581220 PMC7314845

[ref60] Weber M. Liedtke J. Plattes S. Lipski A. (2019). Bacterial community composition of biofilms in milking machines of two dairy farms assessed by a combination of culture-dependent and -independent methods. PLoS One 14:e0222238. doi: 10.1371/journal.pone.0222238, PMID: 31509598 PMC6738651

[ref61] Wit G. D. Svet L. Lories B. Steenackers H. P. (2022). Microbial interspecies interactions and their impact on the emergence and spread of antimicrobial resistance. Annu. Rev. Microbiol. 76, 179–192. doi: 10.1146/annurev-micro-041320-031627, PMID: 35609949

[ref62] Xu Y. Nagy A. Bauchan G. R. Xia X. Nou X. (2017). Enhanced biofilm formation in dual-species culture of listeria monocytogenes and Ralstonia insidiosa. AIMS Microbiol 3, 774–783. doi: 10.3934/microbiol.2017.4.774, PMID: 31294188 PMC6604966

[ref63] Yang X. Wang H. Hrycauk S. Holman D. B. Ells T. C. (2023). Microbial dynamics in mixed-culture biofilms of salmonella typhimurium and Escherichia coli O157:H7 and bacteria surviving sanitation of conveyor belts of meat processing plants. Microorganisms 11:421. doi: 10.3390/microorganisms11020421, PMID: 36838386 PMC9960345

[ref64] Yuan L. Hansen M. F. Røder H. L. Wang N. Burmølle M. He G. (2020). Mixed-species biofilms in the food industry: current knowledge and novel control strategies. Crit. Rev. Food Sci. Nutr. 60, 2277–2293. doi: 10.1080/10408398.2019.1632790, PMID: 31257907

[ref65] Zeinhom M. Hassan G. Salem H. Corke H. (2021). Prevalence and survival of Stenotrophomonas species in milk and dairy products in Egypt. Foodborne Pathog. Dis. 18, 337–345. doi: 10.1089/fpd.2020.2893, PMID: 33625272

