# Supplementary material for: Synergistic interactions in multispecies biofilm combinations of bacterial isolates recovered from diverse food processing industries
Source: Front Microbiol. 2023 Apr 13;14:1159434. doi: 10.3389/fmicb.2023.1159434 (PMC10133454; doi:10.3389/fmicb.2023.1159434)
Supplement: Supplementary file 2 [file Image_1.pdf]

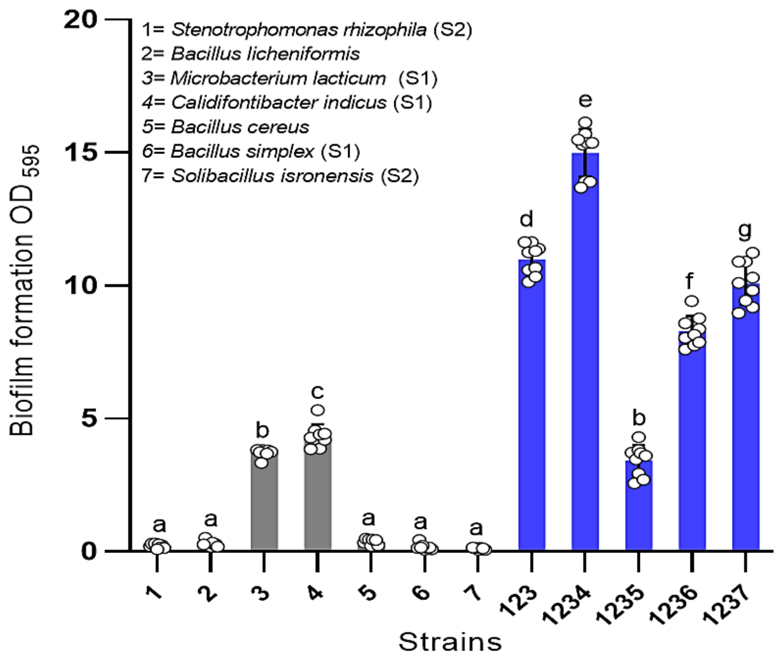

Figure S1. Differential interaction of *Calidifontibacter indicus*, *Bacillus cereus*, *Bacillus simplex*, and *Solibacillus isorensis* as the fourth species with a three-species combination comprising *Stenotrophomonas rhizophila*, *Bacillus licheniformis*, and *Microbacterium lacticum*. It indicated attenuation of synergy by the addition of *B. cereus* as the fourth species. Biofilm mass of the three-species community has also been provided as reference that helps to assess how the addition of the fourth species affects development of the synergy. For comparison purposes, biofilm formation by all strains in single culture is indicated in the grey bars. Comparisons were made using one-way ANOVA using with Duncan's post hoc test ($p < 0.05$ for significance).The presentation shown above is an example that is based on some data from four-species combinations of dairy group 3 strains.
